# D-Dimer Testing for the Exclusion of Pulmonary Embolism Among Hospitalized Patients With COVID-19

**DOI:** 10.1001/jamanetworkopen.2021.28802

**Published:** 2021-10-08

**Authors:** Constantine N. Logothetis, Thomas A. Weppelmann, Aryanna Jordan, Catherine Hanna, Sherry Zhang, Shaun Charkowick, Asa Oxner

**Affiliations:** 1 Department of Internal Medicine, Morsani College of Medicine, University of South Florida, Tampa; 2 Morsani College of Medicine, University of South Florida, Tampa

## Abstract

This prognostic study evaluates the use of plasma D-dimer concentrations to rule out pulmonary embolism among patients hospitalized with COVID-19.

## Introduction

For more than 2 decades, the plasma D-dimer assay has been used in conjunction with clinical prediction scores to rule out pulmonary embolism (PE) among patients with a low pretest probability of having this condition without the need for more costly and invasive methods.^1,2^ The increased thrombotic risk among patients hospitalized with COVID-19 (ie, those with high pretest probability of PE) and increased D-dimer levels in the absence of thrombosis diverge considerably from the study population used to originally validate this assay.^3^ However, the availability of D-dimer samples routinely collected from patients hospitalized with COVID-19 and the heterogeneity of early, smaller studies generated uncertainty regarding the clinical utility of the assay in this setting.^4^ Therefore, we conducted a diagnostic accuracy study to characterize the performance of D-dimer using various threshold values to exclude PE among patients hospitalized with COVID-19.

## Methods

This diagnostic study was approved by the University of South Florida institutional review board, which granted a waiver of informed consent because this study analyzed deidentified medical records. This study used the Standards for Reporting of Diagnostic Accuracy (STARD) reporting guideline for diagnostic studies.

A retrospective, cross-sectional sample of 1541 patients consecutively hospitalized with COVID-19 at a single hospital from January 1, 2020, to February 5, 2021, was collected. Plasma D-dimer concentrations from an automated, standardized assay (expressed as fibrinogen equivalent units) were compared with the criterion standard of computed tomographic pulmonary angiography (CTPA) among 287 patients with suspected PE. D-dimer distributions among patients with and without PE were compared using descriptive statistics and linear regression (after log-normal transformation at α = .05). The ability of plasma D-dimer concentrations collected the day of CTPA to correctly classify patients with PE was evaluated with a static threshold of 0.5 μg/mL or more (to convert to nanomoles per liter, multiply by 5.476) and an age-adjusted threshold (ie, D-dimer value, 0.01 × [age − 50 years]) for individuals aged older than 50 years.^5^ Receiver operator characteristic curves (ROCs) were evaluated for thresholds and an expanded set of D-dimer values ranging from 0.5 μg/mL to 20 μg/mL. Data were analyzed using Stata statistical software version 13 (StataCorp) from May through August 2021.

## Results

Among 287 patients with COVID-19 and suspected PE (177 [51.4%] men; mean [SD] age, 58.2 [16.1] years), 118 patients required intensive care unit levels of care (41.1%) and 27 patients died during hospitalization (9.4%). Of 287 patients with CTPA, 37 patients had radiographic evidence of PE (12.9%) and 250 patients did not (87.1%); 265 patients had plasma D-dimer levels of 0.05 μg/mL or more (92.3%), including all patients with PE and 225 of 250 patients without PE (91.2%). The median (IQR) D-dimer values were 1.0 (0.6-1.8) μg/mL for 250 patients without PE and 6.1 (2.0-19.4) μg/mL for 37 patients with PE. D-dimer values ranged from 0.2 μg/mL to 128 μg/mL among patients without PE and from 0.5 μg/mL to more than 10 000 μg/mL among patients with PE, and patients without PE had statistically significantly decreased mean (SD) D-dimer values (8.7 [11.6] μg/mL vs 1.2 [2.8] μg/mL; *P* < .001).

A D-dimer concentration of 0.05 μg/mL was associated with a sensitivity of 100%, specificity of 8.8%, negative predictive value (NPV) of 100%, positive predictive value (PPV) of 13.9%, and negative likelihood ratio (NLR) of less than 0.1. The age-adjusted threshold was associated with a sensitivity of 94.6%, specificity of 22.8%, NPV of 96.6%, PPV of 13.9%, and NLR of 0.24. The ROC analyses (Figure) yielded areas under the curve that were not statistically significantly different using the 0.5 μg/mL threshold compared with the age-specific threshold (0.81 vs 0.80; *P* = .67). Performance measures for thresholds 0.5 μg/mL (sensitivity, 100%; specificity, 9.3%; accuracy, 21.0%; positive likelihood ratio [PLR], 1.10; NLR, 0) to 20 μg/mL (sensitivity, 20.0%; specificity, 96.6%; accuracy, 86.7%; PLR, 5.90; NLR, 0.57) are presented in the Table.

**Figure.**
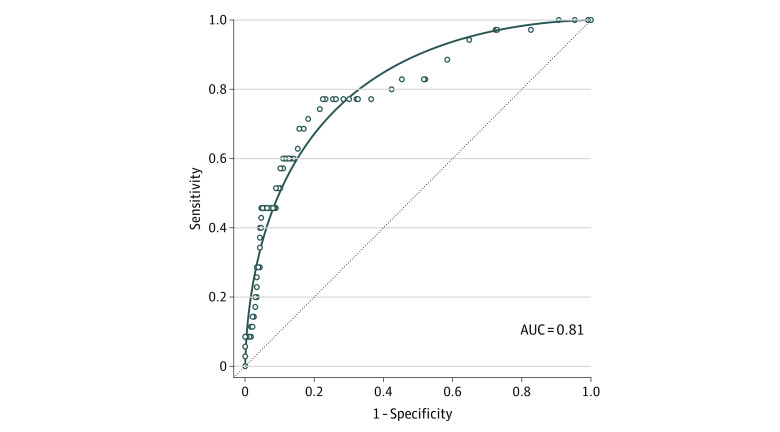
Receiver Operator Characteristic Curve for Plasma D-Dimer Concentrations in μg/mL The receiver operator characteristic curve of plasma D-dimer concentration to classify pulmonary embolism diagnosed by computed tomographic pulmonary angiography at all values of plasma D-dimer in micrograms per milliliter is presented. The performance characteristics at all observed D-dimer concentrations (blue dots), a smoothed, parametric estimation function (solid line), and an uninformative hypothetical test (dashed line) are presented. AUC indicates area under the curve.

**Table.** Performance Measures of Plasma D-Dimer Over Range of Cutoff Points

D-dimer cutoff point, μg/mL	Performance, %^a^			PLR	NLR
	Sensitivity	Specificity	Accuracy		
0.5	100	9.3	21.0	1.10	0
0.6	97.1	17.4	27.7	1.18	0.16
0.7	97.1	27.5	36.5	1.34	0.10
0.8	94.3	35.2	42.8	1.45	0.16
0.9	88.6	41.5	47.6	1.51	0.28
1.0	82.9	48.3	52.8	1.60	0.35
1.5	77.1	69.9	70.9	2.56	0.33
2.0	77.1	77.5	77.5	3.44	0.29
2.5	62.9	84.8	81.9	4.12	0.44
5.0	51.4	90.3	85.2	5.28	0.47
10.0	45.7	94.9	88.6	8.99	0.54
20.0	20.0	96.6	86.7	5.90	0.57

Abbreviations: NLR, negative likelihood ratio; PLR, positive likelihood ratio.

SI conversion factors: To convert micrograms to milliliter to nanomoles per liter, multiply by 5.476.

^a^ The performance measures of a plasma D-dimer assay to classify pulmonary embolism diagnosed by computed tomographic pulmonary angiography with classification cutoff points from 0.5 μg/mL to 20 μg/mL are presented.

## Discussion

This diagnostic study found that all hospitalized patients with COVID-19 and radiographic evidence of PE had plasma D-dimer levels of 0.05 μg/mL or greater. If using D-dimer to exclude patients with PE, the increased values we found among 92.3% of patients suggest that this assay would be less useful than in the populations in which it was originally validated, among which a minority of patients had increased D-dimer values. Setting higher D-dimer thresholds was associated with improved specificity at the cost of an increased false-negative rate that could be associated with an unacceptable patient safety risk. The inclusion of patients with D-dimer and CTPA results was necessary to estimate diagnostic performance; however, this may have introduced selection bias by excluding patients unable to undergo CTPA. Nonetheless, given the high pretest probability of PE and low specificity observed in this and other studies,^6^ these results suggest that the use of D-dimer levels to exclude PE among patients hospitalized with COVID-19 may be inappropriate and have limited clinical utility.
